# GO FEAT: a rapid web-based functional annotation tool for genomic and transcriptomic data

**DOI:** 10.1038/s41598-018-20211-9

**Published:** 2018-01-29

**Authors:** Fabricio Almeida Araujo, Debmalya Barh, Artur Silva, Luis Guimarães, Rommel Thiago Juca Ramos

**Affiliations:** 1 Universidade Federal do Pará, Instituto de Ciências Biológicas, Rua Augusto Corrêa, 01 - Guamá, Belém, PA Brazil; 2 Centre for Genomics and Applied Gene Technology, Institute of Integrative Omics and Applied Biotechnology (IIOAB), Nonakuri, Purba Medinipur, WB-721172 India

## Abstract

Downstream analysis of genomic and transcriptomic sequence data is often executed by functional annotation that can be performed by various bioinformatics tools and biological databases. However, a full fast integrated tool is not available for such analysis. Besides, the current available software is not able to produce analytic lists of annotations and graphs to help users in evaluating the output results. Therefore, we present the Gene Ontology Functional Enrichment Annotation Tool (GO FEAT), a free web platform for functional annotation and enrichment of genomic and transcriptomic data based on sequence homology search. The analysis can be customized and visualized as per users’ needs and specifications. GO FEAT is freely available at http://computationalbiology.ufpa.br/gofeat/ and its source code is hosted at https://github.com/fabriciopa/gofeat.

## Introduction

Giving biological meaning to genomic and transcriptomic data is laborious and time consuming, especially considering the large amount of data generated by high-throughput technologies^1^ and the number of tools, web-servers and databases developed for this purpose^2^. The biological analysis is often given by functional annotation through Gene Ontology (GO) database^3^ which is widely used as the gene functions dictionary. Besides, it’s very usual to perform data functional enrichment by the integration of several databases such as: UniProt^4^, InterPro^5^, KEGG^6^, Pfam^7^, NCBI^8^ and SEED^9^.

Many tools are available for the annotation process: Blast2GO^10^, AmiGO^11^, GOrilla^12^, REVIGO^13^, QuickGO^14^, NaviGO^15^. However, these tools have limitations: a) not all are completely and freely available; b) installation, configuration and command line are complex; c) lack of visual interface; d) limited capacity or sequence number limitation for analysis e) difficulty to share and export results. To address these issues, we developed GO FEAT, a free, on-line, user friendly platform for functional annotation and enrichment of genomic and transcriptomic data based on sequence homology search, allowing users to export the results to different output formats, to generate reports, tables, GO charts and graphs that help them with downstream analysis.

## Methods

GO FEAT is developed in PHP as back-end programming language. HTML5, CSS3 and JavaScript are used as front-end programming language, and PERL is adapted for remote connection scripts. To store the records from the tool we used MySQL RDBMS. All remote calling is made by public REST API (EMBL-EBI’s public API for Blast, UniProt for database integration, QuickGO for ontologies and SEED’s public API for SEED). The user can share their data to other users, export data to several formats, and generate Gene Ontology charts (general and by type of ontology).

**Figure 1:**
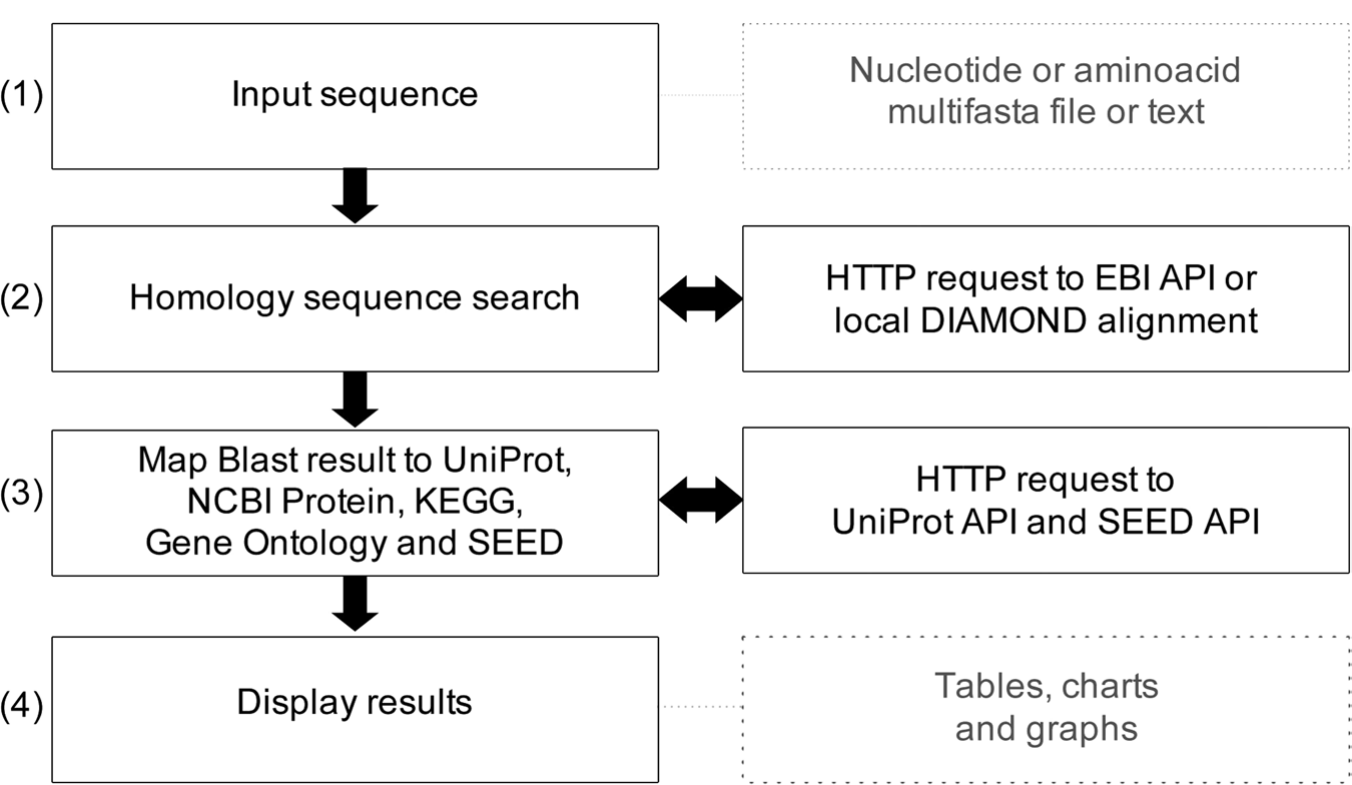
GO FEAT pipeline steps. (1) A multi-fasta file containing any number of sequences (nucleotide or protein) is used as input. (2) Each sequence is used as query against EBI database through EBI public API or local DIAMOND. (3) The alignment results are mapped to UniProt, NCBI Protein, KEGG, GO databases by UniProt public API and SEED database by SEED public API. Finally, (4) the results are displayed in tables, charts and graphs.

GO FEAT receives a multi-fasta file (nucleotide or protein) as an input, once a project is registered or assigned. The pipeline (Fig. 1) proceed to search for homology with e-value defined by the user and then annotate the homologs using public databases. After the submission, each sequence is queued to the processing line. The processing starts with the remote BLAST^16^ using the EMBL-EBI public API^17^ or local DIAMOND^18^ aligner. GO FEAT automatically identifies the type of sequence to be searched (nucleotide or protein) and runs the specific program: BLASTx for nucleotide sequences or BLASTp for protein sequences. The next step is to integrate the result from the alignment to UniProt, NCBI Protein, KEGG, InterPro, Pfam and Gene Ontology databases via UniProt public API and SEED database via SEED public API. After the integration, the results are processed and displayed in graphs, charts, and tables to simplify the analysis.

Since the EBI servers restrict the number of request to 30 at time, a queue control parameter was developed to optimize the server’s resources. For projects with 100 or less sequences, resources are allocated dynamically for maximum of 10 users simultaneously (3 requests for each project). If resources are available, the projects can receive more than 3 requests. Projects with more than 100 sequences are put in a queue for local alignment using DIAMOND that process batches of 500 sequences at a time. This allows the server’s resources usage to be optimized and more sequences can be processed at the same time.

To compare the results from GO FEAT with other tools, we performed the functional annotation in six different scenarios: a random sequence with 500 bp from *Escherichia coli*; the full genome of *Escherichia coli* K-12 MG1655 (4140 CDS and average size of 321 bp) [RefSeq NC_000913.3]; the full genome of *Drosophila melanogaster* BDGP6 (30482 CDS and CDS average size of 668 bp) [Assembly GCA_000001215.4]; the full genome of Nostoc sp. PCC 7107 (5237 CDS and CDS average size of 330 bp) [RefSeq NC_019676.1]; the transcriptomic data from *E. coli* response to five different perturbations (4092 CDS and CDS average size of 326 bp);^19^ and the transcriptomic data from *M. tuberculosis* response to macrophages (4076 CDS and CDS average size of 332 bp)^20^. The results of this comparison are shown in the next section.

## Results

### Interface

GO FEAT was developed to be executed in any modern internet browser. Also, it has a clean and easy-to-use graphic interface. It’s not required any kind of installation of any tool or software and users can execute projects without previous registration.

### Project manager

GO FEAT provides a project manager to facilitate the categorization of each analysis performed by registered users. In the project manager, it’s possible to check the project’s progress, export data to several formats and share projects to other people users to avoid running the same project multiple times.

### Reports, charts and graphs

GO FEAT allows different ways for result visualization: spreadsheet reports present sequences in tables corresponding to its Blast result, which are integrated to several databases, to perform searches and export results; it’s also possible to view the results in graphs and charts, which are divided by molecular function, cellular component and biological process. On each one, it is possible to view all GO terms of each category together with the sequences identification. Finally, the user can view the GO terms with its acyclic graph, downloaded through the Quick GO API.

### Benchmarking

For a 500 bp random sequence chosen from *Escherichia coli*’s genome, GO FEAT takes around 4 minutes for full functional annotation and enrichment while Blast2GO takes around 14 minutes for the same sequence. Direct Blast to NCBI website takes around 2 minutes, however, the mapping between the blast result and other databases are not automatically made. At UniProt, the function annotation and enrichment takes around 2 minutes. Since NCBI’s Blast does not perform a full functional annotation and UniProt website has limitations regarding the number of sequences, they will not be included in further analysis. For complete genomes of model organisms, GO FEAT needs around 5 hours for *Escherichia coli* and 30 hours for *Drosophila melanogaster*. For transcriptomic data, 5 hours were required to peform the functional annotation described in the Jozefczuk’s paper and 5 hours to perform the functional annotation described in Rohde’s paper. Blast2GO was unable to perform the full annotation and enrichment of any complete genome or transcriptomic data analysis in less than 10 days. For non-model organism such as Nostoc sp. PCC 7107, around 4 hours is required to finish the processing in GO FEAT. The time varies depending on server loads of the remote APIs. At full load, around 11 hours was necessary to process 10 projects, each one with 1000 different sequences from *Drosophila melanogaster* and CDS average size of 603 bp. Regarding functionalities, GO FEAT presents useful features in comparison to other functional annotation tools (Table 1) and rapidly process the input sequence and generates the results.

**Table 1:** F1) full freely available; F2) online or simple installation; F3) visual interface; F4) unlimited dataset; F5) share project and F6) export data.

Tool	F1	F2	F3	F4	F5	F6
Blast2GO	No	Yes	Yes	Yes	No	Yes
AmiGO	Yes	Yes	Yes	No	No	Yes
GOrilla	Yes	Yes	Yes	No	No	No
QuickGO	Yes	Yes	Yes	No	No	No
NaviGO	Yes	Yes	Yes	No	No	Yes
GO FEAT	Yes	Yes	Yes	Yes	Yes	Yes

### Limitations

GO FEAT was developed to perform functional annotations on previously predicted genes, coding DNA sequences (CDS), open reading frames (ORF) or transcripts. Thus, large sequences, such as full genomes or contigs, are not suitable to be used as inputs in GO FEAT due to size limitation of alignment softwares.

## Conclusions

Functional characterization of biological sequences is a required step in the analysis of biological data. GO FEAT is an annotation platform integrated with several databases which can be used for different datasets, such as: coding sequences identified after gene prediction and sequences produced after new sequence assembly of next-generation sequencing data. The user can share results with collaborators through graphic interface and can export the results to many formats. Since the tool uses API to access various databases, the annotations are based on most recent and updated data from those databases.

We are committed to maintain GO FEAT for at least 2 years and expect to improve its performance as our computational infrastructure grows. For future works, we plan on adding a prediction step before the functional annotation so users can input large sequences, exporting the predicted sequences.
